# In Vitro Antimicrobial Activity of Various Cefoperazone/Sulbactam Products

**DOI:** 10.3390/antibiotics9020077

**Published:** 2020-02-12

**Authors:** Ming-Jen Sheu, Chi-Chung Chen, Ying-Chen Lu, Bo-An Su, Chun-Cheng Zhang, Shu-Shen Wang, Yin-Ching Chuang, Hung-Jen Tang, Chih-Cheng Lai

**Affiliations:** 1 Division of Gastroenterology and Hepatology, Department of Internal Medicine, Chi Mei Medical Center, Tainan 710, Taiwan; m961193@mail.chimei.org.tw; 2 Department of Medicinal Chemistry, Chia Nan University of Pharmacy & Science, Tainan 717, Taiwan; 3 Department of Medical Research; Chi Mei Medical Center; Tainan 710, Taiwan; s1060491@mail.ncyu.edu.tw (C.-C.C.); biolyc@mail.ncyu.edu.tw (Y.-C.L.); 4 Department of Food Science, National Chiayi University, Chiayi 717, Taiwan; 5 Department of Internal Medicine, Chi Mei Medical Center, Tainan 710, Taiwan; d890814@mail.chimei.org.tw (B.-A.S.); cmh7300@mail.chimei.org.tw (S.-S.W.); 6 Department of Internal Medicine, Kaohsiung Veterans General Hospital, Tainan Branch, Tainan 710, Taiwan

**Keywords:** cefoperazone/sulbactam, multidrug-resistant organism, antimicrobial activity

## Abstract

This study aims to assess the in vitro activity of different samples of cefoperazone/sulbactam (CFP/SUL) against multidrug-resistant organisms (MDROs). Clinical isolates of extended-spectrum β-lactamase (ESBL)-*Escherichia coli*, ESBL-*Klebsiella pneumoniae*, carbapenem-resistant *Acinetobacter baumannii* (CR-AB), and carbapenem-resistant *Pseudomonas aeruginosa* (CR-PA) were collected. The minimum inhibitory concentration (MIC) and time-killing methods were used to assess and compare the in vitro activities of different samples of cefoperazone/sulbactam (CFP/SUL) against these MDROs. For ESBL-*E. coli*, ESBL-*K. pneumoniae*, and CR-PA, product C had smaller variations than product A and B (*p* < 0.05). For CR-AB, product B had the largest variation compared to the other two products (*p* < 0.05). In the time-killing studies, significant differences among the products when used at 16/16 µg/mL were noted for ESBL-*E. coli*, ESBL-*K. pneumoniae*, and CR-AB isolates. In conclusion, this study demonstrated the significantly different activity of different products of CFP/SUL against MDROs.

Cefoperazone/sulbactam (CFP/SUL) is a combination of ß-lactam/ß-lactamase inhibitor, which comprises 1 g CFP and 1 g SUL. CFP/SUL is a broad-spectrum antibiotic against commonly encountered gram-positive cocci, gram-negative bacilli and anaerobes [1,2,3,4]. For multidrug-resistant organisms (MDROs), including extended-spectrum β-lactamase (ESBL)-*Escherichia coli*, ESBL-*Klebsiella pneumoniae*, carbapenem-resistant *E. coli*, and carbapenem-resistant *Acinetobacter baumannii* (CR-AB), CFP/SUL continues to exhibit good in vitro activity, and this potency is not influenced by the inoculum size of ESBL-producing organisms [5,6]. Generic products contain the same active pharmaceutical compound but can include different excipients. In addition, the bioequivalence between generic and branded formulations remains a serious concern. This issue has been investigated for several antimicrobial agents, including meropenem, vancomycin, teicoplanin, ciprofloxacin, oxacillin, gentamicin, amoxicillin, piperacillin/sulbactam, and levofloxacin [7,8,9,10,11,12,13,14,15], but the results are inconsistent. To date, no similar investigation has been performed for CFP/SUL. Therefore, this study was conducted to assess the in vitro activity of three different products of CFP/SUL against MDROs, including ESBL-*E. coli*, ESBL-*K. pneumoniae*, CR-AB, and CR-*Pseudomonas aeruginosa* (CR-PA).

## 1. Material and Methods

### 1.1. Manufacturers of Antimicrobial Agents

Four manufacturers of CFP/SUL (1/1) were selected for this study, and all of them were commercially available. Their products were designated as A, B, C, and S (Table 1). The CFP/SUL samples included three parenteral formulations (A, B, and C) and one comparator, S, formulation (USP, United States Pharmacopeial, Rockville, MD, USA) as a reference standard.

### 1.2. Bacterial Isolates

Each thirty clinical isolates of ESBL-producing *E. coli*, ESBL-producing *K. pneumoniae*, CR-PA, and CR-AB were randomly selected from the Department of Bacteriology at Chi Mei Medical Center which were collected with duplicates eliminated during the period 2008–2015. The most common source of these isolates was respiratory tract, followed by urinary tract and intra-abdominal sites. The isolates were stored at −80 °C in Protected Bacterial Preservers (Technical Service Consultants Limited, Heywood, UK) before use. The ESBL-producing isolates were confirmed using the following four antimicrobial disks: cefotaxime, cefotaxime/clavulanic acid, ceftazidime, and ceftazidime/clavulanic acid. An increase in the zone diameter of ≥5 mm for antibiotics tested in combination with clavulanic acid over the diameter when tested alone indicated that the isolate was an ESBL producer. Carbapenem resistance was defined as resistance to doripenem, ertapenem, imipenem, or meropenem, and the carbapenem-resistant phenotype was confirmed by the modified Hodge test. Species confirmation was performed by standard biochemical methods on a VITEK 2 automated system (bioMérieux, Marcy l’Etoile, France) [5].

### 1.3. In Vitro Susceptibility

The minimal inhibitory concentrations (MICs) of each drug for the tested bacterial isolates were measured by the agar dilution method according to the Clinical Laboratory Standards Institute (CLSI) guidelines [16]. All the MIC tests were performed twice and in triplicate. The concentrations of the drug which we used to conduct MIC tests were 0, 1, 1.5, 2, 2.5, 3, 3.5 4, 4.5, 5, 5.5, 6, 6.5, 7, 7.5, 8, 8.5, 9, 9.5, 10, 12, 14, 16, 20, 24, 28, 32, 36, 40, 48, 56, and 64 µg/mL. The MIC was defined as the lowest concentration of antimicrobial agent that completely inhibited the growth of bacteria. For each isolate, any variation in the MIC was calculated as one minus the ratio of MIC CFP/SUL from each company (MIC_A-C_) divided by the MIC (MIC_S_) of USP CFP/SUL [1-(MIC/MICs)] × 100% [14]. The mean variation was calculated as the sum of variations for the same organism divided by the number of the same organism. QC strains including *E. coli* ATCC 25922, and *P. aeruginosa* ATCC 27853 were used according to the CLSI guideline [17].

### 1.4. Time-Kill Method

Each fifteen ESBL-*E. coli*, ESBL-*K. pneumoniae*, CR-AB, and CR-PA isolates were randomly selected from the clinical isolates. The time-killing method has been described in a previous study [18]. In brief, bacterial suspensions were diluted to 5.0 × 10^5^ colony-forming units (CFUs)/mL in fresh Mueller–Hinton broth. Drug concentrations of CFP/SUL were used at 16/16 µg/mL and 32/32 µg/mL and the susceptible and intermediate breakpoint of cefoperazone, according to CLSI guidelines [17]. Bacterial counts were measured at 4, 8, and 24 h by enumerating the colonies in 10-fold serially diluted specimens of 100-µL aliquots plated on nutrient agar (Difco Laboratories, Sparks, MD) at 37 °C. All the experiments were performed in duplicate.

## 2. Statistical Analysis

A paired *t*-test was used for statistical analysis. The *p*-value for statistical significance for all the analyses was defined as *p* < 0.05.

## 3. Results

### 3.1. MIC of CFP/SUL from Each Formulation

**Figure 1:**
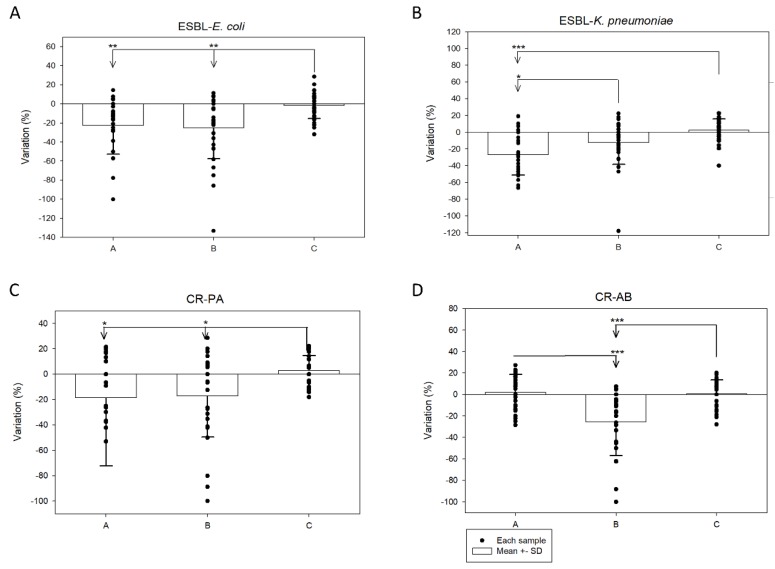
MIC variation in different cefoperazone/sulbactam products against extended-spectrum β-lactamase (ESBL)-*E. coli* (**A**), ESBL-*K. pneumoniae* (**B**), carbapenem-resistant *P. aeruginosa* (**C**), and carbapenem-resistant *A. baumannii* (**D**) (* *p* value < 0.05, ** *p* value < 0.001, *** *p* value < 0.0001).

For ESBL-*E. coli* and ESBL-*K. pneumonia*, carbapenem, tigecycline, colistin, and amikacin showed potent in vitro activity of low MIC levels, however, for CR-PA and CR-AB, only colistin preserved low MIC level (Supplemental Table S1). For CFP/SUL, the MIC ranges and mean variations in the three formulations (product A–C) compared with USP CFP/SUL are presented in Table 2 and Figure 1. For each formula, the MIC_50_ and MIC_90_ were highest for CR-PA, followed by CR-AB. The MIC_50_ and MIC_90_ were lowest for ESBL-*E. coli* and ESBL-*K. pneumoniae*. For both ESBL-*E. coli* and *K. pneumoniae*, product C had the smallest variations compared with product A and B (all *p* < 0.05). For CR-PA, product C had the smallest variation compared with product A and B (*p* < 0.05). For CR-AB, product B had the largest variation compared with the other two formulas (*p* < 0.05).

### 3.2. Time-Killing Method

**Figure 2:**
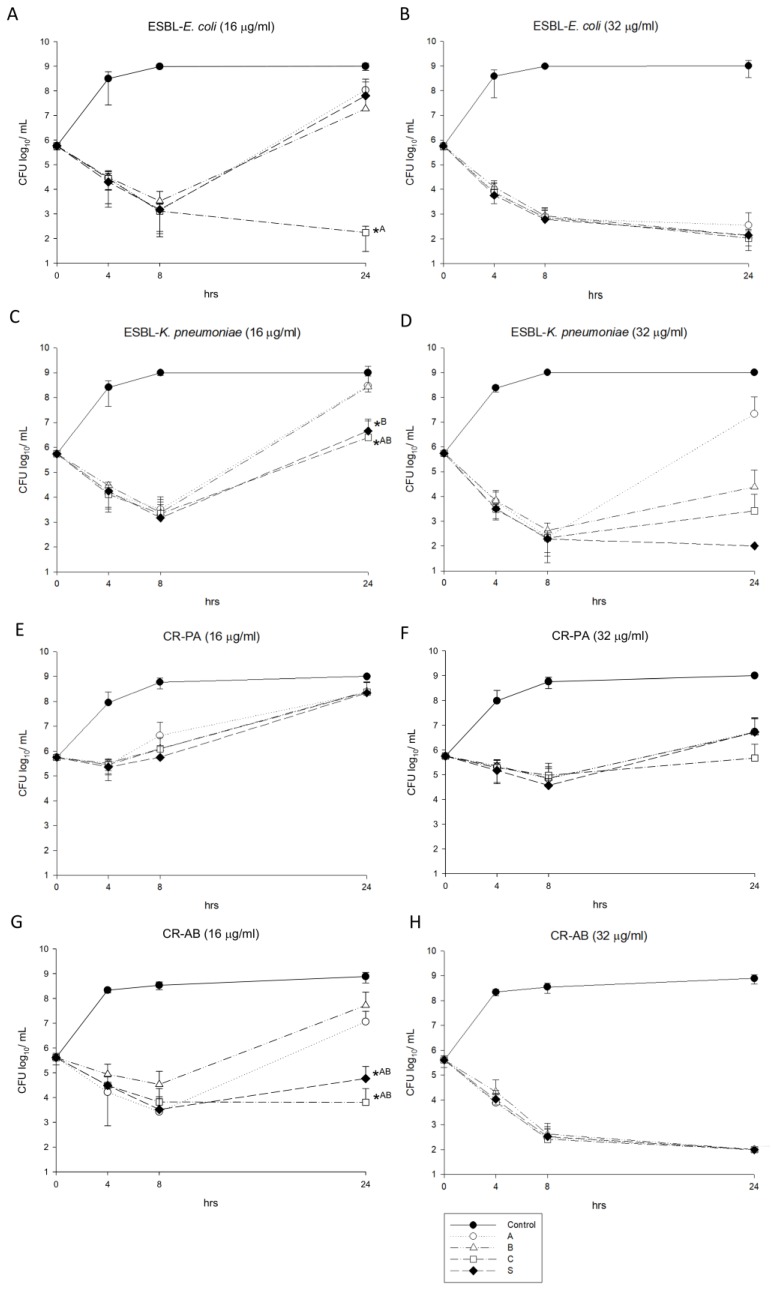
Time-killing study of different cefoperazone/sulbactam products against extended-spectrum β-lactamase (ESBL)-*E. coli* (**A**,**B**), ESBL-*K. pneumoniae* (**C**,**D**), carbapenem-resistant *P. aeruginosa* (**E**,**F**) and carbapenem-resistant *A. baumannii* (**G**,**H**) at the concentrations 16/16 µg/mL and 32/32 µg/mL (* *p* value < 0.05).

For the time-killing studies using CFP/SUL 16/16 µg/mL and 32/32 µg/mL, the bacterial loads are shown in Figure 2. For the ESBL-*E. coli* isolates, product C had greater potency than product A at the concentration 16/16 µg/mL (*p* < 0.05) (Figure 2A), but there were no differences between each isolate at the concentration 32/32 µg/mL (Figure 2B). For the ESBL-*K. pneumoniae* isolates under CFP/SUL using a concentration of 16/16 µg/mL, product C had greater potency than product A and B, and product S had greater potency than product B (both *p* < 0.05) (Figure 2C). In contrast, no difference was found between each formula using the concentration 32/32 µg/mL (Figure 2D). For the CR-PA isolates, no significant difference was observed among the formulas (Figure 2E,F). For the CR-AB isolates, product A and B were less potent than product C at the concentration 16/16 µg/mL (*p* < 0.05) (Figure 2G), but all the three formulas had similar activity at the concentration 32/32 µg/mL (Figure 2H).

## 4. Discussion

This first study compared the in vitro activity of different products of CFP/SUL against four commonly encountered MDROs, including ESBL-*E. coli*, ESBL-*K. pneumoniae*, CR-PA, and CR-AB and found significantly different activity between these products using MICs and time-killing methods. Using the MIC method, variations in the MIC between product A, B, C, and USP CFP/SUL were observed, and these varied according to different MDROs. Using the time-killing method, different potencies between various products were observed, particularly at 16 µg/mL CFP/SUL. In contrast, no significant difference was found using CFP/SUL concentration 32 µg/mL. This result may be due to the high concentration of CFP/SUL overcoming the deficiency of products with decreasing activity. Overall, our findings should raise concerns regarding the noted variation and decreasing in vitro activity in different CFP/SUL products, particularly in this era of increasing use of generic antibiotics for the treatment of acute bacterial infections.

The differences in in vitro activity between generic and brand-name antimicrobial agents have been reported in previous studies [10,14,15] Sun et al. [14] showed that compared with the three generic levofloxacin formulas, brand-name levofloxacin had a similar MIC range to USP levofloxacin RS and had the smallest mean variation (−25% to +13%) compared with USP levofloxacin RS. However, the mean variations in the three generic levofloxacin formulas could be 0% to −50%, −160% to +25%, and−50% to +19%, respectively. Jone et al. [15] demonstrated an average decrease of 16% activity for 23 tested piperacillin/tazobactam generic lots compared with the brand-name product. Fujimura et al. [10] showed that the potency of generic vancomycin and teicoplanin is lower than that of the branded drugs by 14.6% and 17.3%, respectively. Our findings regarding CFP/SUL are consistent with those of previous studies [10,14,15] and indicate that the in vitro activity of antimicrobial agents could vary according to different generic formulas. Although in vitro activity cannot mimic in vivo responses, the range of in vitro activity for different generic products could be a serious concern for clinicians treating severe infections caused by MDROs, such as ESBL-producing organisms or carbapenem-resistant organisms. For these severe MDRO-associated infections, the appropriate choice of antibiotic is essential, and generic antibiotics with decreasing antibacterial activity may have a negative impact on patient outcomes.

This study had two limitations. First, we only did the in vitro tests in this study. However, in vitro activity cannot accurately predict in vivo responses. The different in vitro activity may not equal to different in vivo response. Further in vivo experiment is warranted. Second, we did not investigate the mechanisms causing different in vitro activity in this study. Several factors, including solubility, distribution of active ingredients, and content uniformity between each generic product could affect their in vitro activity. Further detailed study is needed to find out possible mechanism.

## 5. Conclusions

This study demonstrated the significantly different in vitro activities of products of CFP/SUL against MDROs including ESBL-*E. coli*, ESBL-*K. pneumoniae*, CR-PA, and CR-AB using MIC and time-killing methods. Further study is warranted to evaluate the in vivo activity of these formulas.

## Figures and Tables

**Table 1:** List of three cefoperazone/sulbactam products.

Generic No.	Brand Name	Manufacturer	Lot No.	Expiration Date	Vial Strength
A	Cefoperazone sodium and sulbactam sodium	Qilu Antibiotics Pharmaceutical Co., LTD.	8J0199A51	September 2020	1 g
B	Cefoperazone sodium and sulbactam sodium	Shandong Luoxin Pharmaceutical group Hengxin Pharmaceutical Co., LTD.	319032006	March 2021	1 g
C	Brosym	TTY Biopharm Co., LTD	J002846	November 2020	1 g
S	Cefoperazone sodium and sulbactam sodium	US Pharmacopeia	R05230 R03980		0.2 g 0.25 g

**Table 2:** Minimum inhibitory concentration (MIC) results of all cefoperazone/sulbactam formulas against extended-spectrum β-lactamase (ESBL)-*E. coli*, ESBL-*K. pneumoniae*, carbapenem-resistant *A. baumannii*, and carbapenem-resistant *Pseudomonas aeruginosa.*

Bacteria	ESBL-*E. coli*	ESBL-*K. pneumoniae*	Carbapenem-Resistant *P. aeruginosa*	Carbapenem-Resistant *A. baumannii*
Product A				
MIC range	1.38–6.00	2.38–36.00	5.55–61.33	4.00–22.00
MIC_50_	3.63	5.00	22.00	5.50
MIC_90_	4.63	11.00	52.00	11.00
Mean variation ± SD (%)	−22.6 ± 29.9	−27.0 ± 24.0	−18.7 ± 53.4	2.0 ± 16.7
Product B				
MIC range	1.63–6.50	2.25–61.00	5.00–54.00	4.25–22.00
MIC_50_	4.00	4.25	20.00	7.00
MIC_90_	5.88	9.00	48.00	18.00
Mean variation ± SD (%)	−25.1 ± 32.3	−12.3 ± 26.1	−17.1 ± 32.5	−25.4 ± 31.6
Product C				
MIC range	0.88–6.50	2.50–26.00	4.75–40.00	4.00–22.00
MIC_50_	3.25	3.75	20.00	5.50
MIC_90_	4.38	7.50	36.00	11.00
Mean variation ± SD (%)	−1.7 ± 13.6	−2.5 ± 13.3	3.0 ± 11.7	0.7 ± 12.7
Product S				
MIC range	0.88–6.50	2.38–28.00	4.75–38.00	4.00–20.00
MIC_50_	3.13	3.88	20.00	6.00
MIC_90_	4.50	9.00	34.00	9.50
Mean variation ± SD (%)	ND	ND	ND	ND

## Supplementary Materials

The following are available online at https://www.mdpi.com/2079-6382/9/2/77/s1, Table S1: MIC results of antibiotics against extended-spectrum β-lactamase (ESBL)-*E. coli*, ESBL-*K. pneumoniae*, carbapenem-resistant *A. baumannii*, and carbapenem-resistant *Pseudomonas aeruginosa.*
