# Supplementary material for: In Vitro Antimicrobial Activity of Various Cefoperazone/Sulbactam Products
Source: Antibiotics (Basel). 2020 Feb 12;9(2):77. doi: 10.3390/antibiotics9020077 (PMC7168170; doi:10.3390/antibiotics9020077)
Supplement: Supplementary file 1 [file antibiotics-09-00077-s001.pdf]

**Supplemental Table 1.** MIC results of antibiotics against extended-spectrum β-lactamase (ESBL)-*E. coli*, ESBL-*K. pneumoniae,* carbapenem-resistant *A. baumannii,* and carbapenem-resistant *Pseudomonas aeruginosa.*

| | **ESBL-*E.coli*** | | **ESBL-*K.pneumoniae*** | | **Carbapenem-resistant *P.aeruginosa*** | | **Carbapenem-resistant *A.baumannii*** | |
|---|---|---|---|---|---|---|---|---|
| | MIC50 | MIC90 | MIC50 | MIC90 | MIC50 | MIC90 | MIC50 | MIC90 |
| Amikacin | 4 | 8 | 2 | >128 | 8 | >128 | ND | ND |
| Gentamicin | 64 | >128 | 128 | 128 | ND | ND | >128 | >128 |
| Colistin | 1 | 1 | 0.5 | 1 | 2 | 4 | 1 | 4 |
| Doxicycline | 8 | 64 | 64 | >128 | ND | ND | ND | ND |
| Tigecycline | 0.25 | 1 | 1 | 16 | ND | ND | ND | ND |
| Doripenem | 0.063 | 0.125 | 0.062 | 0.25 | 1 | 16 | 64 | >64 |
| Ertapenem | 0.063 | 0.25 | 0.25 | 0.5 | ND | ND | ND | ND |

ND, not done.
